# Harnessing the potential of community-based participatory research approaches in bipolar disorder

**DOI:** 10.1186/s40345-016-0045-5

**Published:** 2016-02-09

**Authors:** Erin E. Michalak, Steven Jones, Fiona Lobban, Guillermo Perez Algorta, Steven J. Barnes, Lesley Berk, Michael Berk, Rachelle Hole, Sara Lapsley, Victoria Maxwell, Roumen Milev, John McManamy, Greg Murray, Mauricio Tohen, Samson Tse, Manuel Sanchez de Carmona, Sheri L. Johnson, The ISBD Taskforce on Community Engagement, CREST.BD

**Affiliations:** Mood Disorders Centre, Department of Psychiatry, University of British Columbia, 2255 Wesbrook Mall, Vancouver, BC V6T 2A1 Canada; Spectrum Centre for Mental Health Research, Division of Health Research, Faculty of Health and Medicine, Lancaster University, Lancaster, UK; Department of Psychology, University of British Columbia, Vancouver, Canada; IMPACT Strategic Research Centre, Faculty of Health, Deakin University, Geelong, Australia; Department of Psychiatry, University of Melbourne, Melbourne, VIC Australia; IMPACT Strategic Research Centre, Deakin University, Melbourne, Australia; Department of Psychiatry, Orygen Research Centre, and the Florey Institute for Neuroscience and Mental Health, University of Melbourne, Melbourne, VIC Australia; Centre for Inclusion and Citizenship, School of Social Work, The University of British Columbia, Vancouver, Canada; Department of Education, University of British Columbia, Vancouver, Canada; Crazy for Life Co., Sechelt, BC Canada; Departments of Psychiatry and Psychology, Queen’s University, Kingston, ON Canada; The Bipolar Expert Series, Alpine, CA USA; Faculty of Life and Social Sciences, Swinburne University of Technology, Hawthorn, Australia; Department of Psychiatry, Health Sciences Center, University of New Mexico, Albuquerque, NM USA; Department of Social Work and Social Administration, The University of Hong Kong, Hong Kong, China; Bipolar Connection Clinic, Mexico City, Mexico; Department of Psychology, University of California, Berkeley, CA USA

**Keywords:** Bipolar disorder, Community-based participatory research, Research methods, Knowledge translation

## Abstract

**Background:**

Despite the rapid growth in the sophistication of research on bipolar disorder (BD), the field faces challenges in improving quality of life (QoL) and symptom outcomes, adapting treatments for marginalized communities, and disseminating research insights into real-world practice. Community-based participatory research (CBPR)—research that is conducted as a partnership between researchers and community members—has helped address similar gaps in other health conditions. This paper aims to improve awareness of the potential benefits of CBPR in BD research.

**Methods:**

This paper is a product of the International Society for Bipolar Disorders (ISBD) Taskforce on Community Engagement which includes academic researchers, healthcare providers, people with lived experience of BD, and stakeholders from BD community agencies. Illustrative examples of CBPR in action are provided from two established centres that specialize in community engagement in BD research: the Collaborative RESearch Team to study psychosocial issues in BD (CREST.BD) in Canada, and the Spectrum Centre for Mental Health Research in the United Kingdom.

**Results and discussion:**

We describe the philosophy of CBPR and then introduce four core research areas the BD community has prioritized for research: new treatment approaches, more comprehensive outcome assessments, tackling stigma, and enhanced understanding of positive outcomes. We then describe ways in which CBPR is ideal for advancing each of these research areas and provide specific examples of ways that CBPR has already been successfully applied in these areas. We end by noting potential challenges and mitigation strategies in the application of CBPR in BD research.

**Conclusions:**

We believe that CBPR approaches have significant potential value for the BD research community. The observations and concerns of people with BD, their family members, and supports clearly represent a rich source of information. CBPR approaches provide a collaborative, equitable, empowering orientation to research that builds on the diversity of strengths amongst community stakeholders. Despite the potential merits of this approach, CBPR is as yet not widely used in the BD research field, representing a missed opportunity.

## Background

Research on bipolar disorder (BD) has expanded rapidly in the past decades. Treatment and biological research has become increasingly sophisticated, and many psychosocial predictors of the course of the disorder have been documented. Despite progress, the field faces challenges in improving quality of life (QoL) and symptom outcomes, adapting treatments for minority and marginalized communities, and implementing research gains in community settings.

Researchers in other fields are increasingly employing CBPR approaches to address complex health and social problems (Chen et al. 2010; D’Alonzo 2010; Hollander 2011; Israel et al. 1998), in part driven by the recovery model that calls for consumer and caregiver engagement, as well as mandates to produce pragmatic and applied research. CBPR has been applied successfully across a range of fields, including cancer, indigenous health, and child health (Israel et al. 1998, 2013; Maar et al. 2011; Vaughn et al. 2013). Despite its potential (Davidson et al. 2010), CBPR is rarely adopted in BD research.

In this paper, we argue that CBPR approaches afford a promising, but as yet under-utilized, approach in the BD research field. We begin by describing the philosophy of CBPR and then outline four core research priorities in BD. We then explore how CBPR can facilitate the achievement of these research domains and discuss some specific examples of ways that CBPR has already been successfully applied to critical issues in BD. Nonetheless, there are unique challenges in applying CBPR in the context of BD as compared to other medical conditions. The very nature of BD suggests that frequently, people will experience symptom fluctuations, and managing these well in the research process requires sensitivity and skill. Our view is that learning to address these barriers is an important goal, in that the potential benefits afforded by CBPR far outweigh the complexities of applying this model. Given this, we provide suggestions for addressing the unique challenges of applying CBPR with BD.

Before beginning, we would note that this paper is one example of CBPR. The genesis of this article arose during an international meeting of researchers and community members focused on BD research. After developing a brief outline for the focus of the paper, we collaborated with the International Society of Bipolar Disorders (ISBD) to form an ISBD Taskforce on Community Engagement; taskforce membership includes academic researchers, healthcare providers, people with lived experience of BD and stakeholders from international BD community agencies such as the Depression and Bipolar Support Alliance (DBSA) and the International Bipolar Foundation (IBF). The outline for the article was reviewed in an initial consensus meeting, and then specific authors contributed sections to reflect their particular expertise. The group as a whole then participated in review and editing, to provide a consensus statement on the importance of CBPR in BD research. We believe that the paper has been enriched by the diverse perspectives of an international panel of expert researchers and healthcare providers from multidisciplinary backgrounds working with those with lived experience to achieve a joint position.

## Community-based participatory research

**Fig. 1:**
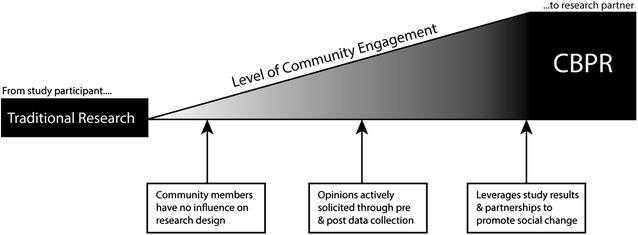
Traditional research and CBPR can be differentiated along a continuum of community involvement

CBPR has been defined as research that is conducted as a partnership between researchers and community members affected by a particular health condition, disability, or issue. It is characterized by substantial community engagement (see Fig. 1) in the development and implementation of the research process, from formulating study goals and hypotheses, to planning the sampling, design, measures, and analyses, to disseminating results (Sciences 2010). Rather than perpetuating the notion of community members as objects of research, the goal is to shape the research process to fit the perspectives and goals of community members (Evans et al. 2009). The emphasis is on generating knowledge that can contribute to community and social change (Israel et al. 1998). Table 1 illustrates some of the many strengths of this approach as adapted from Israel (1998).

**Table 1:** Key principles of CBPR

Recognizes community as central to CBPR
Facilitates collaborative partnerships in all phases of research
Builds on strengths and resources within the community
Gathers knowledge for mutual benefit of all partners
Promotes a co-learning and empowering process that attends to social inequalities
Involves a cyclical and iterative process
Addresses issues from a positive perspective
Integrates biomedical, psychosocial, social, economic, cultural, historical, and political factors as potential determinants of health
Disseminates findings and knowledge gained to all partners

CBPR has been described as a ‘philosophy of engagement’ (Schneider 2012). As a consequence of this orientation and the richer array of questions that may be raised as research targets, openness to adopting diverse methods is prized (Israel et al. 1998). This collaboration may warrant the flexible use of quantitative, qualitative, and mixed-method designs (Khanlou and Peter 1982). That said, it remains paramount for CBPR projects to rely on rigorous research practices and methods (Khanlou and Peter 1982; Greenwood and Levin 2005).

Given that community involvement is central to CBPR, it is important to define community. As used in CBPR, community is not defined in the traditional geopolitical manner, but rather, is based on shared or common characteristics or interests related to the topic of study (Green and Mercer 2001). Community encompasses patients, people who are not receiving medical care, and people within the social support network of the affected individual, including family members, caregivers, significant others, and healthcare providers. Preferred terms for those affected by bipolar symptoms vary substantially. Some use the term consumers, whereas others refer to “service users” for those who take part in mental health services (Shaw 2012). Others refer to lived experience of BD, as many people with BD symptoms are not engaged in mental health treatment (Merikangas et al. 2007). We use these terms interchangeably here.

## Research priorities in BD: a case for the value of CBPR in BD

We begin by broadly describing the types of aspirations that might be accomplished by incorporating CBPR in BD research. After describing these broader aims, we turn to specific examples of CBPR research in BD that has already been accomplished, and concrete findings and gains that have been tackled through this approach.

One of the most important benefits of CBPR is in shaping research to focus on questions that matter to those facing BD. While genetics, neurobiology, and clinical phenomenology are currently the dominant research foci, community members emphasize the need for more pragmatic, real-world studies, the development of new medication and psychological treatments, as well as for research on lifestyle and psychosocial factors (Banfield et al. 2014), prevention (Jacka et al. 2013), and positive features of BD (Lobban et al. 2012a), such as creativity (Johnson et al. 2015a). In the UK, service users have encouraged the development of BD research on recovery, anxiety treatment, and risk factors for suicide (Clements et al. 2013; Jones et al. 2012, 2013a, 2013b). In Canada, research on self-management, stigma, QoL, and psychosocial treatment has been prioritized by CBPR initiatives (Michalak et al. 2012).

Here, we further explore four of the areas prioritized for research by the BD communities engaged with the CREST.BD and Spectrum Centre groups: New treatment approaches, more comprehensive outcome assessments, stigma reduction, and enhanced understanding of positive outcomes. We note ways in which CBPR is ideal for enhancing each of these core priorities.

## New treatment approaches

CBPR could help address a broad range of treatment development and refinement goals in BD. The condition is related to severe outcomes, including high rates of hospitalization, incarceration, suicide, and premature medical morbidity (Goodwin and Jamison 2007), suggesting the need for better treatments. Within the realm of medication treatments, people with lived experience of BD report that current treatment trial designs may not address their core questions, such as “If I don’t take medications or want to take a lower dose than is typically recommended, what are my odds of relapse?” A core issue in treatment research concerns high rates of non-adherence with BD treatments. Here again, CBPR has the potential to be illuminating. Although much of the research literature focuses on issues such as side effects and lack of insight as drivers of poor adherence, people with BD often cease medication use because they did not find that treatment sufficiently improved (or indeed perceived that it has impeded) their functioning or attainment of long-term goals, or due to psychosocial concerns (e.g. impact on identity and stigma issues). This suggests the need for treatment trials that target functional and QoL outcomes.

CBPR approaches have also been used to evolve the research base on self-management strategies that those with BD use to protect against the onset of mania, and empirical research has shown that such strategies can predict lower symptoms of BD over time (Lam and Wong 2005; Lobban et al. 2010). CREST.BD’s CBPR work has identified a host of other self-management strategies, such as regulating sleep, diet and exercise, creative approaches to symptom monitoring and reflective practices, and approaches for improving QoL and interpersonal support around symptoms; these strategies warrant empirical testing. Comparably, in large-scale studies, family members suggest that available psychoeducation programmes stop short of covering the complexity and depth of issues that are invoked by BD, and future programmes may need to personalize recommendations (Berk et al. 2013). We note here that treatment initiatives that focus on peer-support models may offer considerable promise, but further rigorous research is required in this area broadly (Lloyd-Evans et al. 2014; Chinman et al. 2014) and in the BD research field specifically. Taken together, then, CBPR could enrich the nature of research questions regarding treatment, and could help identify new treatment targets and approaches for future research.

## More comprehensive understanding of treatment outcome goals

The goal of delivering more consumer-centered research models will be supported by the development of more comprehensive outcome assessments for BD. With a strong influence from disease models, symptoms have long been a primary outcome measure within BD research. People with lived experience, however, may weigh other outcomes as more important than symptom reduction, such as subjective experiences, QoL (Michalak et al. 2006, 2012; Murray and Michalak 2012), or recovery (Tse et al. 2014; Jones et al. 2015).

The World Health Organization defines QoL as “an individual’s perception of their position in life in the context of the culture and value systems in which they live and in relation to their goals, expectations, standards and concerns” (World Health Organization 1995). QoL encompasses subjective experience, context, and meaning making. Recovery is not synonymous with QoL or symptom reduction but rather refers to the ability to have a meaningful and fulfilling life despite the restrictions of BD.

There are several reasons to consider recovery specifically within the context of BD. In BD, symptoms account for just some of the variance in QoL and functioning. BD is often associated with positive features, and many in the community are interested in achieving a satisfying life in the context of BD more than in “removing” BD symptoms. Some research has examined how consumers perceive the term recovery within BD. In a recent CBPR project, CREST.BD team members used multiple modes of community engagement to improve understanding of recovery, including contracting a peer researcher to help design a community engagement day and embedded qualitative research project. Findings from one study indicated that people with BD living in Vancouver, Canada did not favor the word ‘recovery’ in BD and pointed to discrepancies in how this term is used between affected individuals as compared to their family members (Hou et al. 2012). Similar multi-method research conducted by the Spectrum group, however, has suggested that the term recovery resonates with people with BD in the UK (Todd et al. 2012; Jones et al. 2013b). In sum, research suggests that the term recovery may not be universally accepted in part because misunderstandings can arise in applying this term, and in particular with the distinction between clinical and functional/personal recovery. Regardless of the nuanced definition of recovery, CBPR approaches hold the potential to contribute to recovery. Below, we discuss the development of specific treatment approaches as well as measures concerning recovery and QoL.

## Tackling stigma

Bipolar disorder is heavily stigmatized even amongst mental health conditions, with particularly negative judgements towards manic symptoms such as grandiosity, hyper-sexuality, and aggressive behaviour (Hawke et al. 2013). Stigma is prevalent amongst those affected by BD and their caregivers. CBPR approaches are well-suited for working with socially disadvantaged or marginalized communities, whose members often experience limited access to general and healthcare resources and lower social standing (Johnson and Johnson 2014). The philosophy of engagement endorsed by CBPR (where research is “done with” rather than “done to” people with lived experience) has powerful implications for how they are perceived in society. CBPR challenges the lower social status often associated with BD by bringing members of the community into leadership roles, potentially diminishing traditional power imbalances between researchers and consumers. Peer researchers are often required to develop new skills, and this process may enhance self-efficacy, and occupational or social opportunities.

## Enhanced understanding of positive qualities associated with BD

Beyond the clear need for new research approaches, better outcome measures, and diminished stigma, CBPR has potential to improve understanding of the strengths intrinsic to BD. Clinical research naturally tends to focus on identifying problems, their causes, and their treatments, and this has been the case in BD research, and yet many with lived experience of BD report experiencing considerable benefits from the condition (Murray and Johnson 2010; Seal et al. 2008). Kay Redfield Jamison, an academic and clinician with lived experience of BD, was one of the first to document such benefits. In surveys, those with lived experience endorsed valuing emotional sensitivity, alertness, productivity, social engagement, sexual enjoyment, and creativity as correlates of the disorder. A review of other research has extended this list to include heightened spirituality, empathy, realism, and resilience (Galvez et al. 2011). One of the fascinating qualities of this condition is high rates of accomplishment and creativity amongst those with milder forms of BD and their family members. Recent CBPR highlights that predictors of creativity and positive outcomes may not be those that have been the central focus of research to date; listening to consumers and their family members could reshape a research agenda focused on how to maximize these positive outcomes. We consider this work as we describe examples of concrete gains that have been accomplished by applying CBPR approaches to BD below.

## Illustrative examples of successful application to CBPR in the BD field

We draw on the experiences of two well-established centres for CBPR in BD to provide illustrative examples. The CREST.BD group in Canada (Michalak et al. 2012, 2015) and the Spectrum Centre in the UK are BD specialty centres grounded in community engagement. Both groups routinely involve community members at all phases of the research design, including as grant co-applicants. Both centres have capitalized on the input of those with BD to achieve gains in understanding key priorities for understanding and treating BD and have achieved substantial success in garnering grant support for research on those priorities. Next, we will illustrate how CREST.BD and Spectrum have successfully applied CBPR approaches in the four BD research priority areas outlined above (i.e. treatment approaches, outcome assessments, stigma reduction, and understanding positive outcomes).

### Improved treatment approaches

Traditionally, interventions have been developed based on the expert clinical and academic knowledge. This approach has important limitations as it is unclear to what extent such interventions tackle problems prioritized by individuals living with BD and their relatives. CBPR can help identify key treatment targets, such as anxiety and recovery within BD, that are prioritized by individuals living with BD and their relatives. Service users and carers have then contributed to the development of a wide range of highly face valid interventions for people with lived experience and carers, including novel online interventions, intensive individual therapies, and group programmes (for example, (Jones et al. 2013a, 2015; Lobban et al. 2013, 2015; Morriss et al. 2011; Todd et al. 2014).

For example, positive results of a novel online intervention for improving QoL in late stage BD have recently been published. ORBIT (online, recovery-focused bipolar individual therapy) was developed in close collaboration with the CREST.BD Community Advisory Group, responding to growing doubts about effectiveness of interventions for people in the late stage of BD (more than 10 episodes), and evidence that mindfulness and contemplative approaches may be effective for addressing anxiety within BD. ORBIT aims to specifically improve QoL by developing skills of mindfulness, acceptance, and self-compassion to improve emotion regulation, sleep, and self-concept in late stage BD. The intervention is particularly promising, given evidence that people in late stage BD may not benefit from traditional relapse-prevention focused psychological therapies. A full-scale international randomized controlled trial of the ORBIT intervention is currently underway, with the CREST.BD community advisory group providing a sounding board for developments and refinements. In a study within the Spectrum Centre, CBPR methods were used to develop and test recovery-focused CBT (RfCBT) using an RCT design (Jones et al. 2012). The influence of CBPR in developing RfCBT led to an intervention in which the therapy focus is highly individualized and targeted towards the valued life goals of the client rather than the priorities of the clinician.

CBPR also enhanced recruitment, retention, and therapy engagement across these trials, which were co-designed by people with lived experience, by ensuring communication with participants was targeted, engaging, and provided the information they most commonly requested. This diversity of therapy options reflects the clear message from those with lived experience that their needs are heterogeneous and that these needs must be reflected in the choices offered to people seeking care.

### More comprehensive outcome assessments

As noted above, symptoms have long been a primary outcome measure within BD. Patients, however, often weight recovery or QoL as equally or more important than symptom reduction. A CBPR model was used across all study phases to develop the first bipolar-specific QoL scale (the Quality of Life in Bipolar Disorder Scale, QoL.BD), which emphasizes subjective experience and meaning-making along with other more traditional domains of functioning (Michalak and Murray 2010). The QoL.BD is more sensitive to change in this population than commonly used generic QoL measures (Michalak and Murray 2010). Similar CBPR methods were applied in the iterative development of the first bipolar-specific recovery measure, the Bipolar Recovery Questionnaire (Jones et al. 2013b) which has also proved sensitive to change and has been widely adopted in clinical practice partly driven by high rates of acceptance from service users.

### Stigma

Drawing on community consultations indicating that stigma was a priority BD research area, the CREST.BD (Michalak et al. 2012) team implemented a targeted community engagement event on stigma, including focus groups to discuss definitions, experiences, and subjective impact of stigma as well as to discuss possible stigma reduction interventions. People with BD collaborated on the design and implementation of the research project and were co-authors on the resulting peer-reviewed publication (Suto et al. 2012). Findings from the event were used to secure funding for a project to examine whether a theatre performance could help reduce stigma. One of the grant co-investigators, an actress and mental health educator with lived experience of BD, produced, and performed a one-hour, one-woman theatrical performance entitled ‘That’s Just Crazy Talk’ in which the narrator described her experiences of personal and familial mental illness and related stigma, and her attempts to come to terms with her complex illness. A Community Advisory Group guided the development, implementation, and evaluation of the performance. Findings revealed that the performance lowered stigma amongst healthcare providers as well as people with BD (Michalak et al. 2014). A filmed version of the performance was found to diminish negative attitudes in healthcare providers. Over 7000 people have now seen the performance live. The recorded version of the performance has been adopted into official curricula by post-secondary nursing programmes (e.g. Queen’s University), professional bodies (e.g. National Society of Genetic Counselors), and the Mental Health Commission of Canada’s (MHCC) Opening Minds program—the largest systematic effort in Canadian history focused on reducing mental illness stigma.

### Enhanced understanding of positive qualities associated with BD

Work by the Spectrum group has shown that many people with BD value the positive aspects of their bipolar-related experiences (Lobban et al. 2012b). Three important themes emerged: (1) Direct positive impact of BD experiences on everyday life including amplification of internal states, enhanced abilities, and more intense human connectedness; (2) Lucky to be bipolar—the sense of having been given a special gift; (3) Relationship between the self and bipolar experiences. This kind of in-depth analysis of lived experiences may help us to understand ambivalence to current treatment and to develop interventions that minimize the negative impacts, while recognizing and potentially retaining some of the positives. To improve understanding of the mechanisms guiding elevated rates of creativity in BD, CREST.BD sponsored a community engagement day for those with BD who were engaged in creative professions, and again, hosted focus groups on the topic (Johnson et al. 2015b). Although the literature in this area has tended to focus on a small number of mechanisms, such as potential benefits of BD to divergent thinking, energy, or ambition, the affected individuals suggested a much broader range of potential mechanisms, such as the ability to use rich life experiences as a base for novels and the motivation to develop artistic pursuits for political or emotional expression. This community input helped shape new research hypotheses.

## Challenges

Despite the potential value, several challenges should be considered in implementing CBPR approaches. Some key strategies to consider when implementing CBPR in BD research are provided in Table 2.

**Table 2:** Strategies to consider when implementing CBPR in BD research

Concern	Potential solutions
Some types of conclusions and statements may be distressing for those with lived experience	Consider in advance how to include information about protective factors in research designs; consider language in describing findings carefully; give advance warning to consumers about findings that might be on difficult topics, allowing choice about participation; consider whether findings are being presented in the most compassionate manner; plan for ongoing supervision to review and support individual’s response to difficult material
Those with BD may go through symptomatic periods that interfere with productivity	Plan in advance for back-up and recovery time; consider working with teams of individuals rather than relying on a single person
Symptoms may emerge in a way that interfere with privacy or work flow	Develop an understanding in advance of how symptoms will be discussed and managed if they are apparent in the workplace
Some with lived experience may have less scientific background than other team members	Invest in training team members to understand the research process Develop an understanding of key and valued roles that can be well-managed by those with less scientific background
Some with lived experience may have government benefits that be will be jeopardized if they work more than a certain number of hours, and others may prefer to maintain flexible hours	Discuss levels of commitment and constancy in advance, and use this knowledge to plan work roles that will not suffer from part-time or varied time involvement
Organizations may not value lived experience as well as they do scientific experience, leading to the potential for inequality in promotions and career advancement over time	Team leaders need to work at a systematic level to change organizational barriers

Below, we delve deeper into challenges and potential mitigation strategies in two core areas: people and funding.

### People

Just like any group of people, some people with lived experience are more productive, collaborative, or able to contribute to the research processes than others. It might be prudent to put energy into developing relationships with a small number of peer researchers (just as we would with other colleagues), rather than blindly inviting anyone with a passing interest. At the same time, the most disadvantaged may be the best served by CBPR; careful and well thought-out strategies may be required to involve more disenfranchized community members as well as skill matching to tasks, with recognition that different levels and types of contributions can occur from consumers. It is also important to note (and assess at the point of research initiation) that peer researchers often have diverse reasons for engaging in research and different understandings of parameters for success.

Many barriers to participation can be partially circumvented through appropriate training and ongoing support. The research topics under scrutiny and the nature of research findings may be more impactful for people with lived experiences. For example, peer researchers working with CREST.BD found qualitative statements about stigma to be highly distressing. It is also important to consider the incredible variability in how those with BD may feel over time—by definition, BD involves extreme fluctuations in energy, motivation, concentration, and social function, and these fluctuations need to be planned for in thinking about how to build a successful team of collaborators. Team leaders should plan in advance for how they will address emergent symptoms, and how and when to give a person a break should symptoms emerge. Effective strategies for supporting peer researchers with BD in the CREST.BD and Spectrum include: comprehensive training programmes, the appointment of multiple peer researchers who share research tasks to allow for periods of down-time (e.g. CREST.BD currently has three social media interns who live with BD who take collective responsibility for Web 2.0 outputs; the CREST.BD Community Advisory Group is co-chaired by two people with BD; Spectrum employs two relatives to moderate an online toolkit supporting relatives of people with BD or psychosis), provision of both clinical/academic and peer supervision and sensitive skill matching of individuals to tasks.

We also believe that it is important to identify the contributions that are likely to be most successful. For teams focused on basic research, such as biological markers and endophenotypes, researchers may initially question the ability of those with less scientific background to participate. Consumers, though, will often have very meaningful input to offer on how to embed questions of interest in study design, such as given that a person has biological vulnerabilities, are there social or psychological contexts that would be more protective. They also can offer substantive feedback on how to convey findings in a way that offers clarity.

### Funding

Even though we have noted grant agencies that directly support community-engaged research above, funding for CBPR projects raises several challenges. First, it can be a particular challenge to involve users in the grant development process (one method for community inclusion), because many with lived experience will have no formal role within the university and often live with economic circumstances that mandate financial remuneration. Second, when payment for research involvement could threaten disability benefits, other forms of recognition may be important to offer. Third, many with lived experiences will experience periods of illness in which they will be less able to contribute to team activities; grant budgets are often harshly rigid for addressing this need for fluidity. Effective strategies for ensuring continued funding identified by CREST.BD and Spectrum include: funding from diverse sources (e.g. philanthropic, industry or private sector, non-governmental community agencies, collaborative applications with community partners including National Health Services, and smaller awards from host institutions) in order to bridge the gaps between traditional operating grants, therefore avoiding ‘hit and run’ engagement (Michalak et al. 2012) with the bipolar community, and in the case of CREST.BD, ongoing pursual of funding for the network itself as a discrete entity. Involvement of service user researchers in teaching and clinical implementation of interventions can also provide additional financial support.

One positive development is that some funding agencies now reward more patient-centric research approaches. The Patient-Centered Outcomes Research Institute (PCORI) in the US now provides major annual funding for health research that is guided by patients, caregivers, and the broader healthcare community, one notable initiative being the Mood Patient-Powered Research Network. The Vancouver Foundation in Canada funds health research that is grounded in community priorities and conducted in partnership with communities with the goal of fostering sustainable change after project completion. The National Institute for Health Research in the UK requires public/patient involvement for funding. Such changes in funding models will necessarily help to shape the future of consumer engagement in the development and delivery of new models for healthcare research.

## Conclusions

We believe that CBPR approaches have significant potential value for the BD research community. The observations and concerns of people with BD, their family members and supports clearly represent a rich source of information. CBPR approaches provide a collaborative, equitable, empowering orientation to research that builds on the diversity of strengths amongst community stakeholders. Despite the potential merits of this approach, CBPR is as yet not widely used in the BD research. In an age of translational research, CBPR offers opportunity to enhance the applicability of research gains for improved care; BD researchers are encouraged to seek consultation and collaboration to capitalize on the richness of CBPR approaches. Potential barriers in relation to people and funding are not insurmountable with appropriate consultation and collaboration. We encourage researchers, reviewers, and grant agencies to consider the benefits of CBPR approaches, and relish the opportunity to engage in ongoing debate about how best to maximize these benefits.
